# YPEL3 suppresses epithelial–mesenchymal transition and metastasis of nasopharyngeal carcinoma cells through the Wnt/β-catenin signaling pathway

**DOI:** 10.1186/s13046-016-0384-1

**Published:** 2016-07-11

**Authors:** Jian Zhang, Xin Wen, Xian-Yue Ren, Ying-Qin Li, Xin-Ran Tang, Ya-Qin Wang, Qing-Mei He, Xiao-Jing Yang, Ying Sun, Na Liu, Jun Ma

**Affiliations:** Sun Yat-sen University Cancer Center; State Key Laboratory of Oncology in South China, Collaborative Innovation Center of Cancer Medicine, 651 Dongfeng Road East, Guangzhou, People’s Republic of China

**Keywords:** YPEL3, Nasopharyngeal carcinoma, Epithelial–mesenchymal transition, Metastasis, Wnt/β-catenin

## Abstract

**Background:**

Metastasis remains the major cause of death in nasopharyngeal carcinoma (NPC). Yippee-like 3 (YPEL3) plays an important role in tumorigenesis. However, its function and mechanism in NPC has not been systematically explored.

**Methods:**

We evaluated YPEL3 expression in NPC cell lines and tissues using real-time PCR and western blotting. Then, we established NPC cell lines that stably overexpressed YPEL3 and knocked down YPEL3 expression to explore its function in NPC *in vitro* and *in vivo*. Additionally, we investigated the potential mechanism of YPEL3 action by identifying the Wnt/β-catenin signaling pathway downstream genes using western blotting.

**Results:**

YPEL3 was downregulated in NPC cell lines and tissue samples. Ectopic expression of YPEL3 inhibited NPC cell migration and invasion *in vitro*; while silencing of YPEL3 promoted NPC cell migration and invasion. Further study indicated that overexpression of YPEL3 inhibited NPC cell epithelial–mesenchymal transition (EMT) and that silencing it enhanced EMT. Overexpression of YPEL3 suppressed NPC cell lung metastasis *in vivo*. The mechanism study determined that YPEL3 suppressed the expression levels of Wnt/β-catenin signaling pathway downstream genes and the nuclear translocation of β-catenin.

**Conclusions:**

YPEL3 suppresses NPC EMT and metastasis by suppressing the Wnt/β-catenin signaling pathway, which would help better understanding the molecular mechanisms of NPC metastasis and provide novel therapeutic targets for NPC treatment.

## Background

Nasopharyngeal carcinoma (NPC) is a head and neck cancer with a high prevalence rate (20–50 cases per 100,000 people) in South China [1, 2]. Although intensity-modulated radiotherapy and chemotherapy have improved the local control, distant metastasis remains the major cause of death in NPC [3, 4]. Thus, better understanding the mechanisms underlying NPC metastasis is essential for developing novel treatment strategies.

Yippee-like 3 (YPEL3), a protein encoded by the *Drosophila Yippee* gene [5], is a member of the putative zinc finger motif, which contains proteins with a high degree of conservation among the cysteines and histidines [6]. Investigators first identified YPEL3 as SUAP, or small unstable apoptotic protein, which induced IL3 removal in a myeloid precursor cell line [7]. A number of studies have demonstrated that YPEL3 suppresses tumor growth, proliferation and metastasis in several types of cancer, such as in breast tumors [8] and colon tumors [9]. Recent studies have found that murine YPEL3 is related to cell growth inhibition through apoptosis, which is involved in the programmed cell death of murine myeloid precursor cells during differentiation [7, 10]. However, the roles and mechanism of YPEL3 in NPC development and progression remain unclear.

Recent studies indicate that the Wnt/β-catenin signaling pathway is overactivated in several human cancers, including NPC [11–13]. Briefly, Wnt ligand binding initiates the Wnt/β-catenin pathway, and the cytoplasmic degradation complex is inhibited, which leads to T-cell factor/lymphoid enhancer factor activation of the Wnt downstream genes. The Wnt/β-catenin signaling pathway is one of the most important signaling pathways identified as being involved in tumor metastasis [14–17]; however, whether the molecular mechanism of YPEL3 is associated with the pathway and the relevance between YPEL3 and Wnt/β-catenin signaling in NPC remain to be elucidated.

In this study, we investigated the roles and mechanism of YPEL3 in NPC metastasis. We discovered that YPEL3 expression was decreased in NPC cell lines and clinical samples. YPEL3 overexpression inhibited NPC cell invasion and metastasis *in vitro* and *in vivo*. Further studies demonstrated that YPEL3 overexpression inhibited epithelial–mesenchymal transition (EMT) by suppressing the nuclear translocation of β-catenin.

## Methods

### Cell culture and clinical specimens

Human NPC cell lines (CNE-1, CNE-2, SUNE-1, HNE-1, HONE-1, 5-8 F, 6-10B) were cultured in RPMI 1640 medium (Invitrogen, Carlsbad, CA, USA) supplemented with 10 % (v/v) fetal bovine serum (FBS; Gibco, Grand Island, NY, USA). The human immortalized nasopharyngeal epithelial NP69 cell line was grown in keratinocyte serum–free medium (Invitrogen, Carlsbad, CA, USA) supplemented with bovine pituitary extract (BD Biosciences, San Jose, CA, USA). We maintained 293FT cells in Dulbecco’s modified Eagle’s medium (Invitrogen, Carlsbad, CA, USA) supplemented with 10 % FBS. In addition, 22 freshly frozen NPC biopsy samples and 12 normal nasopharyngeal epithelium samples were collected from Sun Yat-sen University Cancer Center. This study was approved by the Institutional Ethical Review Boards of our center, and written informed consent was obtained from each patient.

### RNA isolation and reverse transcription–PCR (RT-PCR)

Total RNA was extracted using TRIzol (Invitrogen, Carlsbad, CA, USA) and quantified at 260 nm by a NanoDrop 2000 spectrophotometer (Thermo Scientific, Waltham, MA, USA). Total RNA (2 μg) was reverse-transcribed to complementary DNA (cDNA) using an RT kit (Promega, Madison, WI, USA). Quantitative PCR was performed in triplicate using Platinum SYBR Green qPCR Super Mix-UDG reagents (Invitrogen, Carlsbad, CA, USA) on a CFX96 Touch™ sequence detection system (Bio-Rad, Hercules, CA, USA). The following primer sequences were used for amplification: *YPEL3* forward, 5′-CCACGACGACCTCATCTC-3′; reverse, 5′-CATATTTCCAGCCCAAAGT-3′; *E-cadherin* forward, 5′-GAAGAGGACCAGG ACTTTGAC-3′; reverse, 5′- GTAGTCATAGTCCTGGTCTTTGTC-3′; *Vimentin* forward, 5′- TCAGACAGGATGTTGACAATGC-3′; reverse, 5′- TCATATTGCTGACGTACGTCAC-3′. *GAPDH* was used as the endogenous control, and the comparative threshold cycle (2^-ΔΔCT^) equation was used to calculate the relative expression levels.

### Western blotting

Cultured cells were washed twice with ice-cold phosphate-buffered saline (PBS), solubilized in a lysis buffer containing 1 mmol/L protease inhibitor cocktail (FDbio Science, Hangzhou, China) on ice, and quantified using the bicinchoninic acid method. Cell lysate protein samples were separated by sodium dodecyl sulfate–polyacrylamide gel electrophoresis and then electrophoretically transferred to polyvinylidene difluoride membranes (Millipore, Billerica, MA, USA). The membranes were blocked with 5 % skim milk in Tris-buffered saline–Tween (TBST) buffer (10 mmol/L Tris–HCl [pH 7.4], 150 mmol/L NaCl, 0.1 % Tween 20) for 2 h. Protein expression was detected following overnight incubation at 4 °C using primary antibodies against HA (1:2000, Sigma-Aldrich, USA); YPEL3 (1:100, Abcam, Cambridge, MA, USA), β-catenin (1:500, Proteintech, Wuhan, China), c-MYC (1:2000, Proteintech, Wuhan, China), cyclin D1 (1:500, Proteintech, Wuhan, China), α-catenin (1:500, BD Biosciences, San Jose, CA, USA), E-cadherin (1:500, BD Biosciences, San Jose, CA, USA), vimentin (1:500, BD Biosciences, San Jose, CA, USA), GSK3β(1:1000, Proteintech, Wuhan, China), TBP (1:800, Proteintech, Wuhan, China), and GAPDH (1:500, Proteintech, Wuhan, China). Thereafter, the membranes were washed and incubated for 1 h at room temperature with the appropriate horseradish peroxidase–conjugated secondary antibody. After the membranes were washed with TBST buffer three times, the proteins were visualized with an enhanced chemiluminescence reagent (Beyotime, Shanghai, China). The bands were analyzed using Image J software.

### Stable cell line establishment and YPEL3 small interfering RNAs (siRNAs)

The pSin-EF2-puro-YPEL3-HA or pSin-EF2-puro-vector plasmids were obtained from Land. Hua Gene Biosciences (Guangzhou, China). All plasmids were verified by DNA sequencing before use; the pSin-EF2-puro-vector plasmid was used as the control. Stably transfected cells were selected using puromycin and were confirmed using quantitative RT-PCR. SiRNA#1 targeting YPEL3-Homo-974 (siYPEL3), which was obtained from GenePharma Co., Ltd (Shanghai, China), was a pool of siRNAs for the *YPEL3* gene (sense strand: 5′-GCCACCUCUUCAACUCAGTT-3′; antisense strand: 5′-CUGAGUUGAAGAG GUAGGCTT-3′); siRNA#2 targeted YPEL3-Homo-838 cDNA (sense strand: 5′-GCGGAU UUCAAAGCCCAAGTT-3′; antisense strand: 5′-CUUGGGUUUGAAUCCGCTT-3′).

### Wound healing assay

CNE-2 and SUNE-1 cells were seeded onto a 6-well culture plate and cultured to a subconfluent state in complete medium. After 24-h starvation in serum-free medium, cell monolayers were linearly scraped with a P-200 pipette tip. Cells that had detached from the bottom of the wells were gently aspirated and incubated in serum-free medium for 24 h. The width of the scratch was monitored under a microscope and quantified in terms of the difference between the original width of the wound and the width after cell migration.

### Transwell migration and invasion assays

Transwell migration and invasion assays were carried out using Transwell chambers (Corning, Tewksbury, MA, USA) with 8-μm pore polyethylene membranes. For the migration assay, cells were placed in the upper chamber of each insert that had not been coated with Matrigel (BD Biosciences, San Jose, CA, USA). For the invasion assay, cells were placed in the upper chamber of inserts that had been pre-coated with Matrigel. Plasmid- or siRNA-transfected CNE-2 and SUNE-1 cells (5 × 10^4^ for the migration assay;1 × 10^5^ for the invasion assay) were added to the upper chamber in serum-free medium, and the lower chamber contained culture medium with 20 % FBS to act as a chemoattractant. The cells were incubated for 12 h or 24 h at 37 °C in 5 % CO_2_, and then they were fixed and stained. Cells on the undersides of the filters were observed and counted under × 200 magnification.

### Immunofluorescence staining

CNE-2 and SUNE-1 cells were cultured on coverslips in 6-well plates, washed with PBS, fixed with 4 % paraformaldehyde for 30 min, and permeabilized with 0.5 % Triton X-100 for 10 min. After blocking with 1 % bovine serum albumin for 1 h, the cells were immunostained with antibody against E-cadherin or vimentin at a 1:500 dilution. The cells were washed with PBS and incubated with Alexa Fluor–conjugated goat anti-mouse secondary antibody (1:200, A11011; Invitrogen). After washing with PBS, the nuclei were stained with DAPI (Invitrogen,) for 15 min, and fluorescence images were obtained using a confocal scanning microscope (OLYMPUS FV1000; Olympus, Tokyo, Japan) and analyzed using Image-Pro Plus 6.0 software.

### Animal experiments

Female, pathogen-free, athymic nude mice were purchased from Charles River Laboratories (Beijing, China) (*n* = 9mice per group). SUNE-1 cells (1 × 10^6^) stably overexpressing vector or YPEL3 that had been resuspended in 200 μL serum-free medium were injected intravenously into the tail veins of the mice. After 8 weeks, the mice were sacrificed and the lung tissues were fixed for calculating the numbers of macrometastatic nodes formed on the surface of lungs. Then, the tissues were paraffin-embedded, serial 5-μm tissue sections were cut, and one of every ten sections was stained with hematoxylin–eosin (HE) for examination of micrometastatic nodes formed in the lungs as previously described [18, 19]. All animal research was conducted in accordance with the detailed rules approved by the Animal Care and Use Ethnic Committee of Sun Yat-sen University Cancer Center and all efforts were made to minimize animal suffering.

### Statistical analysis

All statistical analyses were carried out with SPSS software (standard version 17.0, Chicago, IL, USA). All experiments were performed in three independent experiments and all data are presented as the mean ± SD. Significant differences between two groups were analyzed using two-tailed unpaired Student’s *t*-test and a *P*-value <0.05 was considered statistically significant.

## Results

### YPEL3 is downregulated in NPC cells and tissues

**Fig. 1:**
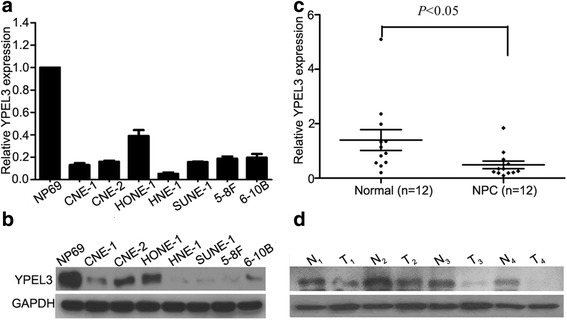
YPEL3 mRNA and protein expression levels in NPC cell lines and tissues. **a-b** Quantitative RT-PCR (**a**) and western blotting analysis (**b**) of YPEL3 expression levels in NPC cell lines. **c** Quantitative RT-PCR analysis of *YPEL3* mRNA expression levels in NPC (*n* = 12) and normal nasopharyngeal epithelial tissues (*n* = 12); **d** western blotting analysis of YPEL3 protein levels in NPC (T, *n* = 4) and normal nasopharyngeal epithelial tissues (N, *n* = 4). All of the experiments were performed at least three times. Data presented are the mean ± SD; the *P*-value was calculated using the Student *t*-test

The mRNA and protein expression levels of YPEL3 in the normal nasopharyngeal epithelial NP69 cell line and seven NPC cell lines were assessed by quantitative RT-PCR and western blotting. Compared to NP69 cells, YPEL3 was significantly downregulated in the NPC cell lines at both mRNA and protein level (Fig. 1a, b). We also investigated the *YPEL3* mRNA levels in 12 frozen NPC tissues and 12 normal nasopharyngeal epithelial tissues, and observed that *YPEL3* mRNA was expressed at considerably lower levels in the NPC tissues (Fig. 1c). Western blotting validated the decreased YPEL3 protein levels in the NPC tissues (Fig. 1d). These results indicate that decreased YPEL3 expression may promote NPC development and progression.

### YPEL3 suppressed NPC cell migration and invasion in vitro

**Fig. 2:**
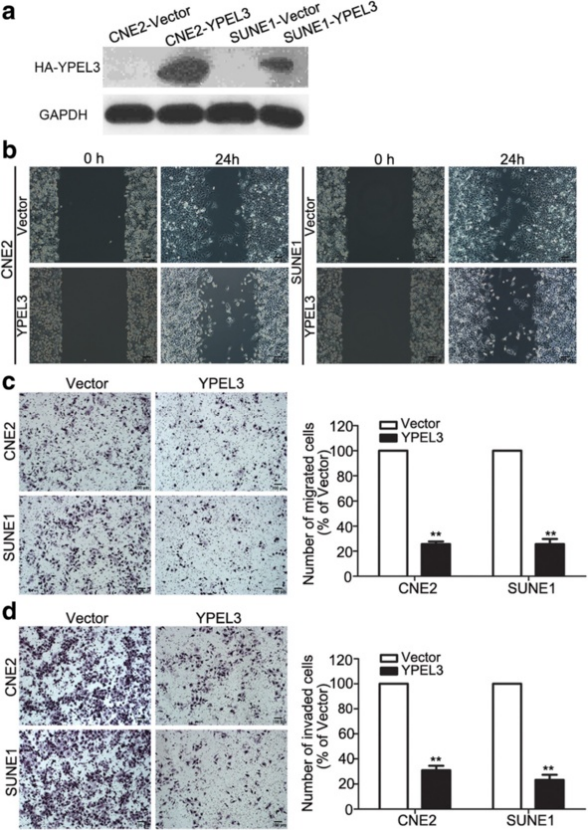
Effects of YPEL3 overexpression on NPC cell migration and invasion *in vitro*. **a** Representative western blotting analysis of YPEL3 overexpression in CNE-2 and SUNE-1 cells. GAPDH served as the loading control. **b-d** Representative images and quantification of the effects of YPEL3 overexpression on the migratory and invasive abilities of CNE-2 and SUNE-1 cells as determined by wound healing (**b**), Transwell migration (**c**), and invasion (**d**) assays. All of the experiments were performed at least three times. Data presented are the mean ± SD; **P < 0.01 compared with control using Student *t*-test

To study whether YPEL3 affects the migratory and invasive abilities of NPC cells, we performed wound healing, Transwell migration, and invasion assays using CNE-2 and SUNE-1 cells stably overexpressing YPEL3 or vector. As shown in Fig. 2a, Western blotting validated that YPEL3 protein level was obviously elevated after stably overexpressing YPEL3 in NPC cells. The wound healing assay demonstrated that the migratory ability of CNE-2 and SUNE-1 cells stably overexpressing YPEL3 was much lower than that of cells expressing vector plasmid (Fig. 2b). Overexpressing YPEL3 suppressed the migratory (Fig. 2c) and invasive (Fig. 2d) abilities of the NPC cells as determined by the Transwell migration and invasion assays. These findings indicate that YPEL3 inhibits the migratory and invasive abilities of NPC cells *in vitro*.

### Silencing YPEL3 promoted NPC cell migration and invasion in vitro

**Fig. 3:**
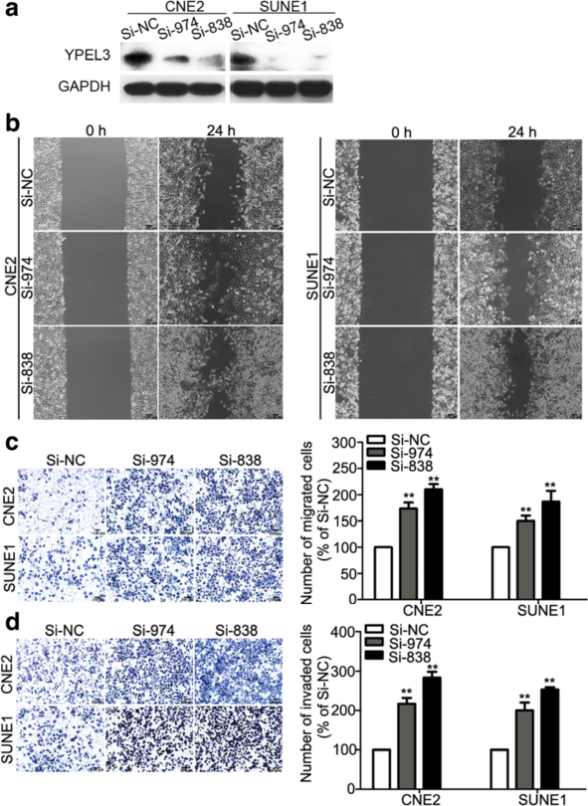
Effects of YPEL3 silencing on NPC cell migration and invasion *in vitro*. **a** Representative western blotting analysis of YPEL3 silencing in CNE-2 and SUNE-1 cells. GAPDH served as the loading control. **b-d** Representative images and quantification of the effects of YPEL3 silencing on the migratory and invasive abilities of CNE-2 and SUNE-1 cells as determined by wound healing (**b**), Transwell migration (**c**), and invasion assays (**d**). All of the experiments were performed at least three times. Data presented are the mean ± SD; **P < 0.01 compared with control using Student *t*-test

To further investigate whether silencing of YPEL3 affects the migratory and invasive abilities of NPC cells, we transiently transfected CNE-2 and SUNE-1 cells with siYPEL3 or control siRNA, and performed wound healing, Transwell migration, and invasion assays. As shown in Fig. 3a, Western blotting confirmed that the YPEL3 protein level was remarkably decreased after silencing of YPEL3 in NPC cells. The wound healing and Transwell migration assays showed that cells transfected with siYPEL3 migrated more slowly than cells transfected with control siRNA (Fig. 3b, c). Knocking down YPEL3 promoted NPC cell invasive capability as determined by the Transwell invasion assay (Fig. 3d). These findings indicate that silencing YPEL3 promotes the migratory and invasive abilities of NPC cells *in vitro*.

### YPEL3 suppressed NPC cell EMT

**Fig. 4:**
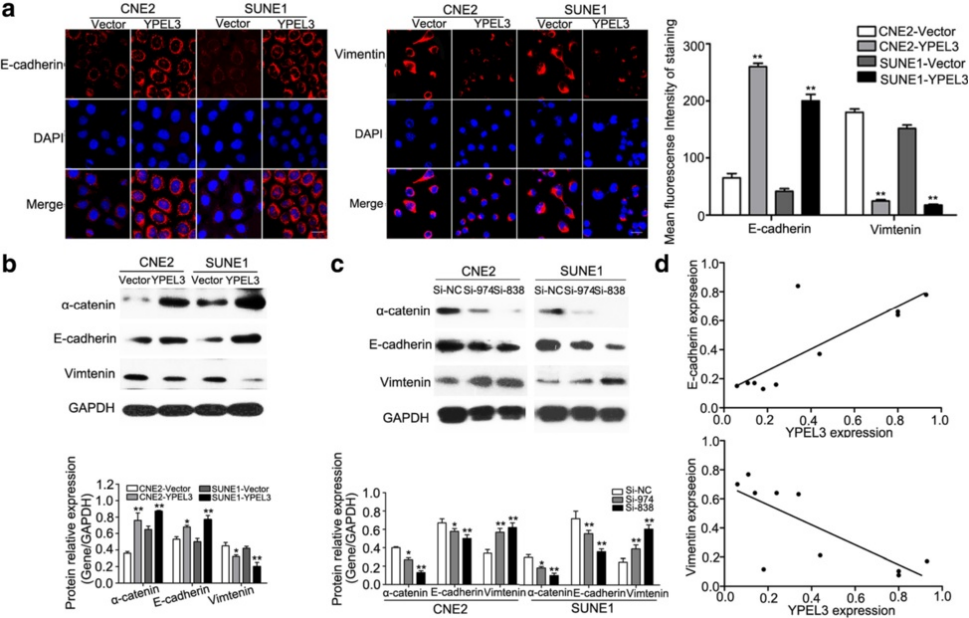
YPEL3 overexpression inhibited EMT and its correlation with EMT markers. **a** Immunofluorescence staining and quantification analysis of E-cadherin and vimentin expression levels (×100). **b, c** Western blot analysis of EMT marker expression levels in NPC cells in which YPEL3 was overexpressed (**b**) or silenced (**c**). **d** Correlations between YPEL3 expression and E-cadherin and vimentin expression in NPC tissue samples (*n* = 10). All of the experiments were performed at least three times. Data presented are the mean ± SD; **P < 0.01, *P < 0.05 compared with control using Student *t*-test

As EMT is important for the acquisition of metastatic potential in tumors, we determined the expression levels of the epithelial markers E-cadherin and α-catenin, and that of the mesenchymal marker vimentin using immunofluorescence staining and western blotting. Immunofluorescence staining revealed that YPEL3 overexpression significantly rescued E-cadherin expression but inhibited vimentin expression (Fig. 4a); western blotting revealed similar changes in epithelial and mesenchymal marker expression in the CNE-2 and SUNE-1 cells overexpressing of YPEL3 (Fig. 4b). Conversely, silencing YPEL3 suppressed E-cadherin and α-catenin expression but increased vimentin expression (Fig. 4c). In addition, Spearman’s correlation analysis showed thatYPEL3 mRNA expression was positively correlated with E-cadherin expression, and inversely correlated with vimentin expression in 10 NPC tissue samples (*r* = 0.803 and *r* = -0.754, respectively; both *p* < 0.001, Fig. 4d). These observations demonstrate that YPEL3 suppresses NPC cell EMT.

### YPEL3 suppressed lung metastasis in vivo

**Fig. 5:**
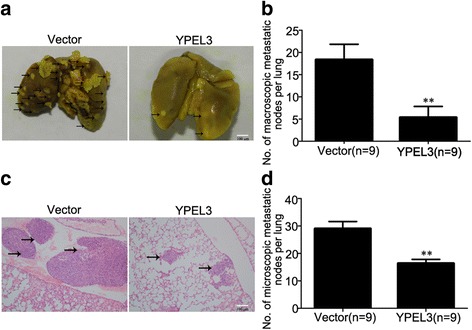
YPEL3 suppressed lung metastasis *in vivo.* Nude BALB/C mice were intravenously injected via the tail vein with SUNE-1 cells stably overexpression YPEL3 or vector (*n* = 9 in each group). **a** Representative images of macroscopic lung metastases, arrowheads indicate the metastatic nodes; **b** Quantification of the average number of macroscopic metastatic nodes formed on the lung surface; **c** Representative images of HE staining (×100); **d** Quantification of the average number of microscopic metastatic nodes formed in the lungs based on pathological analysis of HE-stained sections. Data presented are the mean ± SD; **P < 0.01 compared with control using Student *t*-test

To determine the role of YPEL3 in NPC cell metastasis *in vivo*, we constructed a lung metastasis model by injecting SUNE-1 cells stably overexpressing YPEL3 or vector into the tail veins of nude mice. After 8 weeks, fewer macroscopic metastatic nodes were seen on the lung surfaces of the YPEL3-overexpressing group as compared with the vector group (Fig. 5a, b). In addition, there were smaller and fewer microscopic metastatic nodules in the YPEL3-overexpressing group than in the vector group (Fig. 5c, d). These results indicate that YPEL3 suppresses NPC cell metastasis, suggesting YPEL3 functions as a negative regulator of metastasis.

### YPEL3 inhibits NPC metastasis through the Wnt/β-catenin signaling pathway

**Fig. 6:**
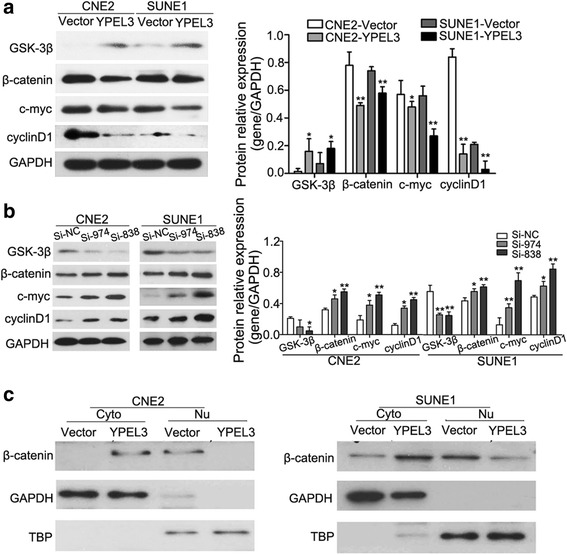
YPEL3 inhibited the Wnt/β-catenin signaling pathway. **a** Representative western blotting and quantification analysis of GSK-3β, β-catenin, c-MYC, and cyclin D1 expression levels after YPEL3 overexpression. **b** Representative western blotting and quantification analysis of GSK-3β, β-catenin, c-MYC, and cyclin D1 expression levels after YPEL3 silencing. **c** YPEL3 inhibited the nuclear (Nu) translocation of β-catenin. Cyto, cytoplasmic. All of the experiments were performed at least three times. Data presented are the mean ± SD; *P < 0.05 and **P < 0.01 compared with control using Student *t*-test

As Wnt/β-catenin plays a critical in EMT induction and maintenance, we examined the protein expression of the Wnt/β-catenin signaling genes for GSK3β, β-catenin, c-MYC, and cyclin D1 in CNE-2 and SUNE-1 cells overexpressing YPEL3 or in which YPEL3 had been silenced to explore the YPEL3 mechanism underlying NPC metastasis. Overexpressing YPEL3 elevated the protein expression of GSK3β and suppressed the protein expression of β-catenin, c-MYC, and cyclin D1(Fig. 6a). Conversely, silencing YPEL3 inhibited the protein expression of GSK3β and increased the protein expression of β-catenin, c-MYC, and cyclin D1 the genes (Fig. 6b). Moreover, subcellular protein fraction study confirmed that YPEL3 overexpression decreased nuclear β-catenin expression and increased cytoplasmic β-catenin expression (Fig. 6c), which indicates that the suppressive effect of YPEL3 may take place through the inhibition of β-catenin nuclear translocation. These data demonstrate that YPEL3 plays a critical role in mediating the Wnt/β-catenin signaling pathway.

## Discussion

In this study, we show for the first time that YPEL3 expression is downregulated in NPC cell lines and tissue samples at both mRNA and protein level. Restoring YPEL3 expression suppressed NPC cell migration and invasion *in vitro* and *in vivo* and reversed EMT by inhibiting the Wnt/β-catenin signaling pathway. In light of these findings, we believe that YPEL3 has the potential to be a novel predictor of distant metastasis as well as a promising therapeutic target in NPC.

NPC has a high rate of local invasion and early metastasis [20–22]; intensity-modulated radiotherapy has greatly improved the local control of NPC, but distant metastasis contributes to the majority of treatment failure and death in patients with NPC [23, 24]. Therefore, it is vital to detect metastasis-associated biomarkers that can effectively distinguish patients with NPC who are at high risk of metastasis, and further develop novel treatment strategies for NPC.

YPEL3, first identified as SUAP, is classified as a tumor suppressor for two reasons. First, its activation led to IL3 removal in a myeloid precursor cell line [7]. Second, its expression is downregulated in many cancers and it plays critical roles in cancer cell proliferation, motility, and apoptosis [7, 8]. In the present study, YPEL3 expression levels were decreased in NPC cell lines and tissue samples at both mRNA and protein level; furthermore, YPEL3 overexpression inhibited SUNE-1 and CNE-2 cell invasion, metastasis, and EMT *in vitro* and *in vivo*. These findings indicate that YPEL3 plays a tumor-suppressive role in NPC, which is consistent with the role of YPEL3 in other cancers [6–8].

EMT contributes to cancer cell invasion, metastatic dissemination, and acquisition of therapeutic resistance [25]. Multiple signaling pathways, including the nuclear factor kappa B (NF-kB), Wnt, transforming growth factor-β (TGF-β), and Notch signaling pathways, are involved in regulating EMT [26–30]. Wnt/β-catenin plays a critical role in EMT induction and maintenance [31, 32]. Dysregulated signaling of the Wnt/β-catenin signaling pathway increases the malignancy of various human cancers, including NPC [33–37]. In this study, we observed that YPEL3 increased membrane-associated β-catenin but decreased nuclear β-catenin. It is possible that YPEL3 decreases the level of Wnt mediators, leading to reduced Wnt activity and β-catenin destruction.

Wnt binding to its membrane receptor activates intracellular signaling and leads to the dissociation of *β*-catenin from the degradation complex consisting of Axin, APC, CK1 and GSK3*β* [38, 39]. β-catenin has been reported to be aberrantly accumulated in human tumors, while GSK-3β can induce ubiquitination and proteasomal degradation of β-catenin [40, 41]. In addition, c-myc expression is thought to be an indicator of Wnt/β-Catenin activity. Therefore, we examined the changes to the downstream target genes of the Wnt/β-catenin signaling pathway by western blotting. YPEL3 overexpression improved GSK-3βexpression levels and suppressed β-catenin, c-MYC, and cyclin D1 expression, whereas silencing YPEL3 increased their expression. The present study confirms that YPEL3 is an important tumor suppressor in NPC and has identified novel YPEL3/Wnt/β-catenin signaling in NPC metastasis.

## Conclusions

In this research, we systematically analyzed YPEL3 function and mechanism in NPC. Given the importance of YPEL3 expression and the Wnt/β-catenin signaling pathway in NPC development and progression, our findings not only provide further understanding of the molecular mechanisms underlying NPC metastasis, but also identify aberrant Wnt/β-catenin signaling as a promising new therapeutic target for NPC. Further research to validate this hypothesis is underway.

## Abbreviations

EMT: epithelial mesenchymal transition
FBS: fetal bovine serum
HE: hematoxylin-eosin staining
MMP: murine myeloid precursor
MMTV: integration site family
NPC: nasopharyngeal carcinoma
PBS: phosphate-buffered saline
RT-PCR: real-time polymerase chain reaction
siRNAs: small interfering RNAs
SUAP: small unstable apoptotic protein
TBST: Tris-buffered saline Tween
TGF-β: transforming growth factor–β
Wnt: wingless-type
YPEL3: Yippee-like-3
