# Correction to: YPEL3 suppresses epithelial–mesenchymal transition and metastasis of nasopharyngeal carcinoma cells through the Wnt/β-catenin signaling pathway

**DOI:** 10.1186/s13046-020-01710-y

**Published:** 2020-10-12

**Authors:** Jian Zhang, Xin Wen, Xian-Yue Ren, Ying-Qin Li, Xin-Ran Tang, Ya-Qin Wang, Qing-Mei He, Xiao-Jing Yang, Ying Sun, Na Liu, Jun Ma

**Affiliations:** Sun Yat-sen University Cancer Center, State Key Laboratory of Oncology in South China, Collaborative Innovation Center of Cancer Medicine, 651 Dongfeng Road East, Guangzhou, People’s Republic of China

**Correction to: J Exp Clin Cancer Res 35, 109 (2016)**

**https://doi.org/10.1186/s13046-016-0384-1**

Following publication of the original article [1], the authors identified that mismatched images had been used in Figs. 2, 3 and 6. Specifically, the following panels have been replaced with corrected images created using the raw study data:

- Fig. 2d: effect of YPEL3 on SUNE-1 cells
- Fig. 3b: Si-974 CNE-2 24 h; Si-803 SUNE-1 0 h
- Fig. 3c: Si-838 CNE-2
- Fig. 3d: Si-974 CNE-2 and Si-974 SUNE-1
- Fig. 6c: GAPDH CNE-2 Nu Vector

The corrected figures are given below. The corrections do not affect the conclusions of the article.

**Fig. 2:**
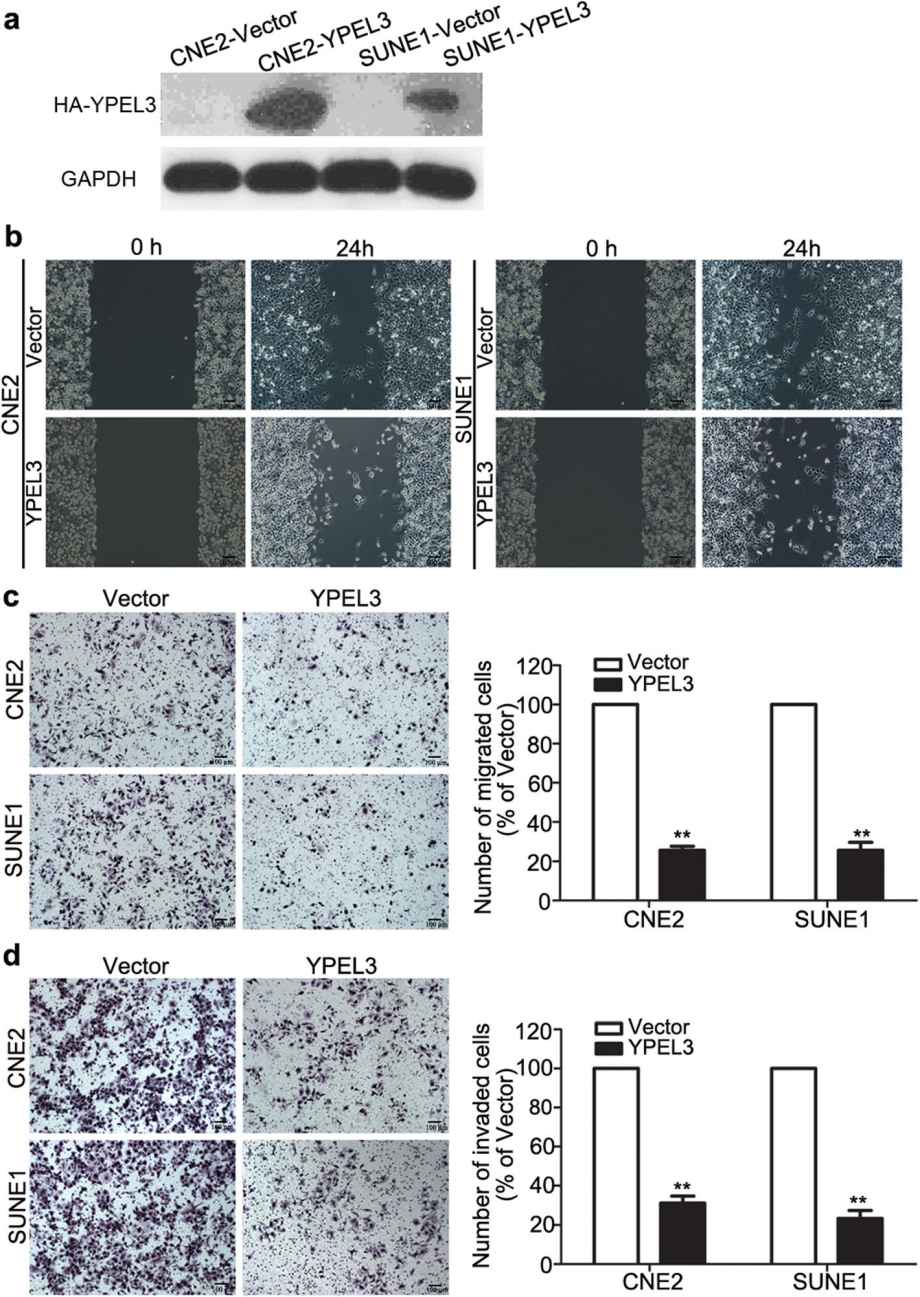
Effects of YPEL3 overexpression on NPC cell migration and invasion *in vitro*. **a** Representative western blotting analysis of YPEL3 overexpression in CNE-2 and SUNE-1 cells. GAPDH served as the loading control. **b-d** Representative images and quantification of the effects of YPEL3 overexpression on the migratory and invasive abilities of CNE-2 and SUNE-1 cells as determined by wound healing (**b**), Transwell migration (**c**), and invasion (**d**) assays. All of the experiments were performed at least three times. Data presented are the mean ± SD; **P < 0.01 compared with control using Student *t*-test

**Fig. 3:**
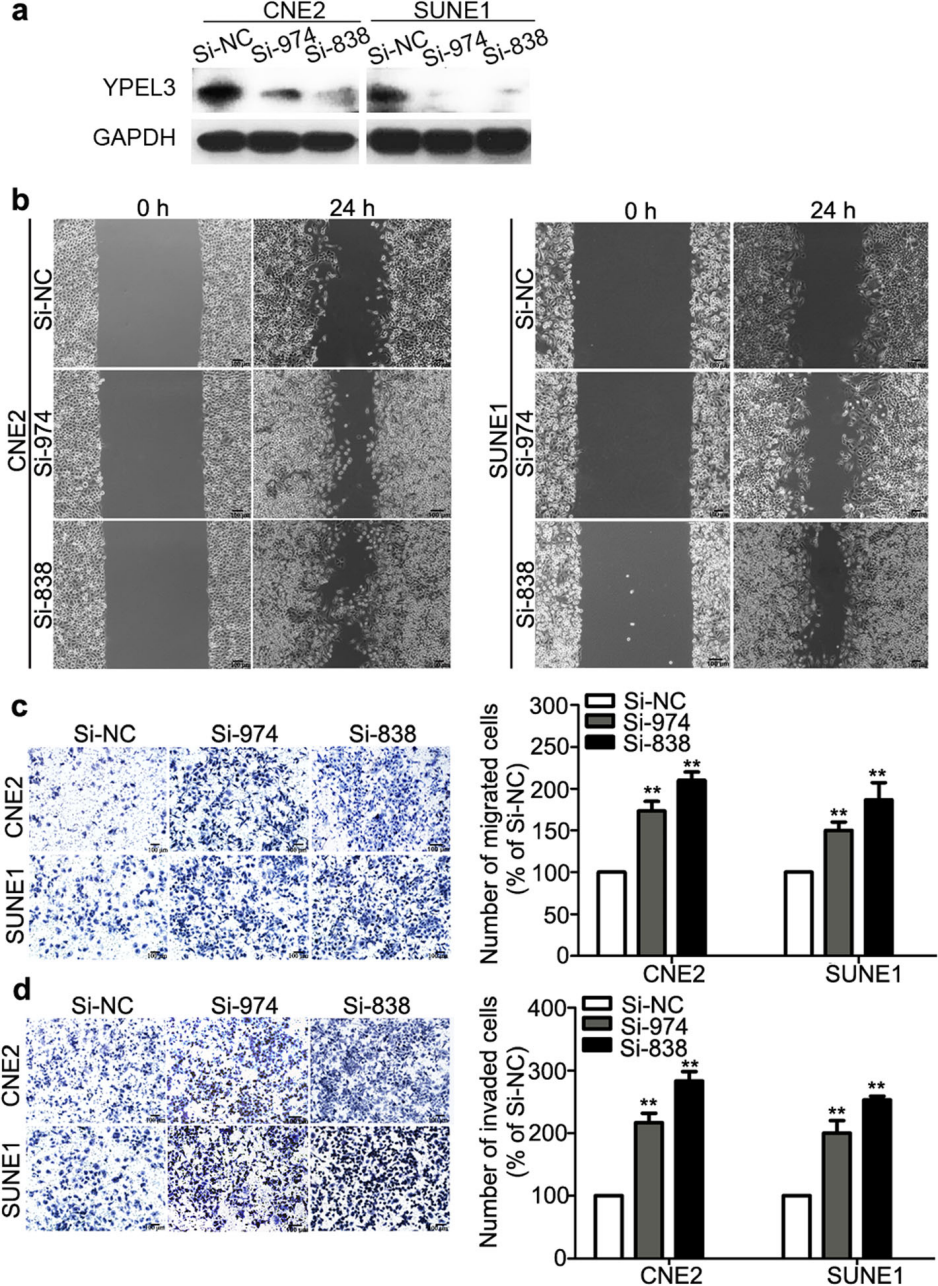
Effects of YPEL3 silencing on NPC cell migration and invasion *in vitro*. **a** Representative western blotting analysis of YPEL3 silencing in CNE-2 and SUNE-1 cells. GAPDH served as the loading control. **b-d** Representative images and quantification of the effects of YPEL3 silencing on the migratory and invasive abilities of CNE-2 and SUNE-1 cells as determined by wound healing (**b**), Transwell migration (**c**), and invasion assays (**d**). All of the experiments were performed at least three times. Data presented are the mean ± SD; **P < 0.01 compared with control using Student *t*-test

**Fig. 6:**
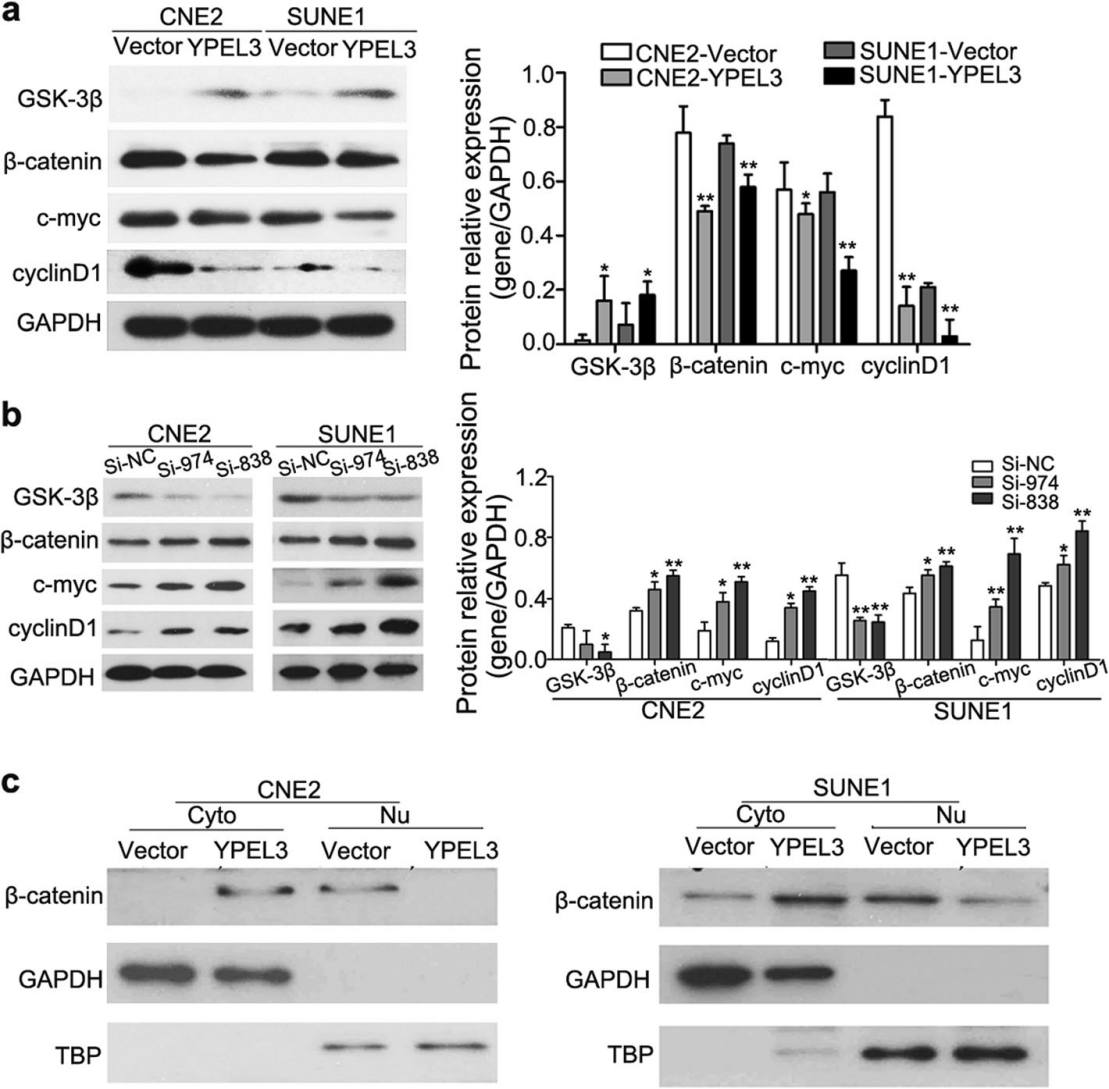
YPEL3 inhibited the Wnt/β-catenin signaling pathway. **a** Representative western blotting and quantification analysis of GSK-3β, β-catenin, c-MYC, and cyclin D1 expression levels after YPEL3 overexpression. **b** Representative western blotting and quantification analysis of GSK-3β, β-catenin, c-MYC, and cyclin D1 expression levels after YPEL3 silencing. **c** YPEL3 inhibited the nuclear (Nu) translocation of β-catenin. Cyto, cytoplasmic. All of the experiments were performed at least three times. Data presented are the mean ± SD; *P < 0.05 and **P < 0.01 compared with control using Student *t*-test
