# Correction to: YPEL3 suppresses epithelial–mesenchymal transition and metastasis of nasopharyngeal carcinoma cells through the Wnt/β-catenin signaling pathway

**DOI:** 10.1186/s13046-021-02189-x

**Published:** 2021-12-23

**Authors:** Jian Zhang, Xin Wen, Xian-Yue Ren, Ying-Qin Li, Xin-Ran Tang, Ya-Qin Wang, Qing-Mei He, Xiao-Jing Yang, Ying Sun, Na Liu, Jun Ma

**Affiliations:** Sun Yat-sen University Cancer Center; State Key Laboratory of Oncology in South China, Collaborative Innovation Center of Cancer Medicine, 651 Dongfeng Road East, Guangzhou, People’s Republic of China

**Correction to: J Exp Clin Cancer Res 35, 109 (2016)**

**https://doi.org/10.1186/s13046-016-0384-1**

Following publication of the original article [1] and subsequent correction [2], the authors identified that further mismatched images were present in the corrected version of Fig. 3. Specifically, in Fig. 3, the SUNE1-si974 cells at 0 h (Si-NC and Si-974) were incorrect, and have been replaced by the correct images.

In addition, the figures 2, 3, and 6 in the previous correction [2] were not replaced in the original article [1], thus, the figure 2 and 6 have been replaced together with figure 3.

**Fig. 2:**
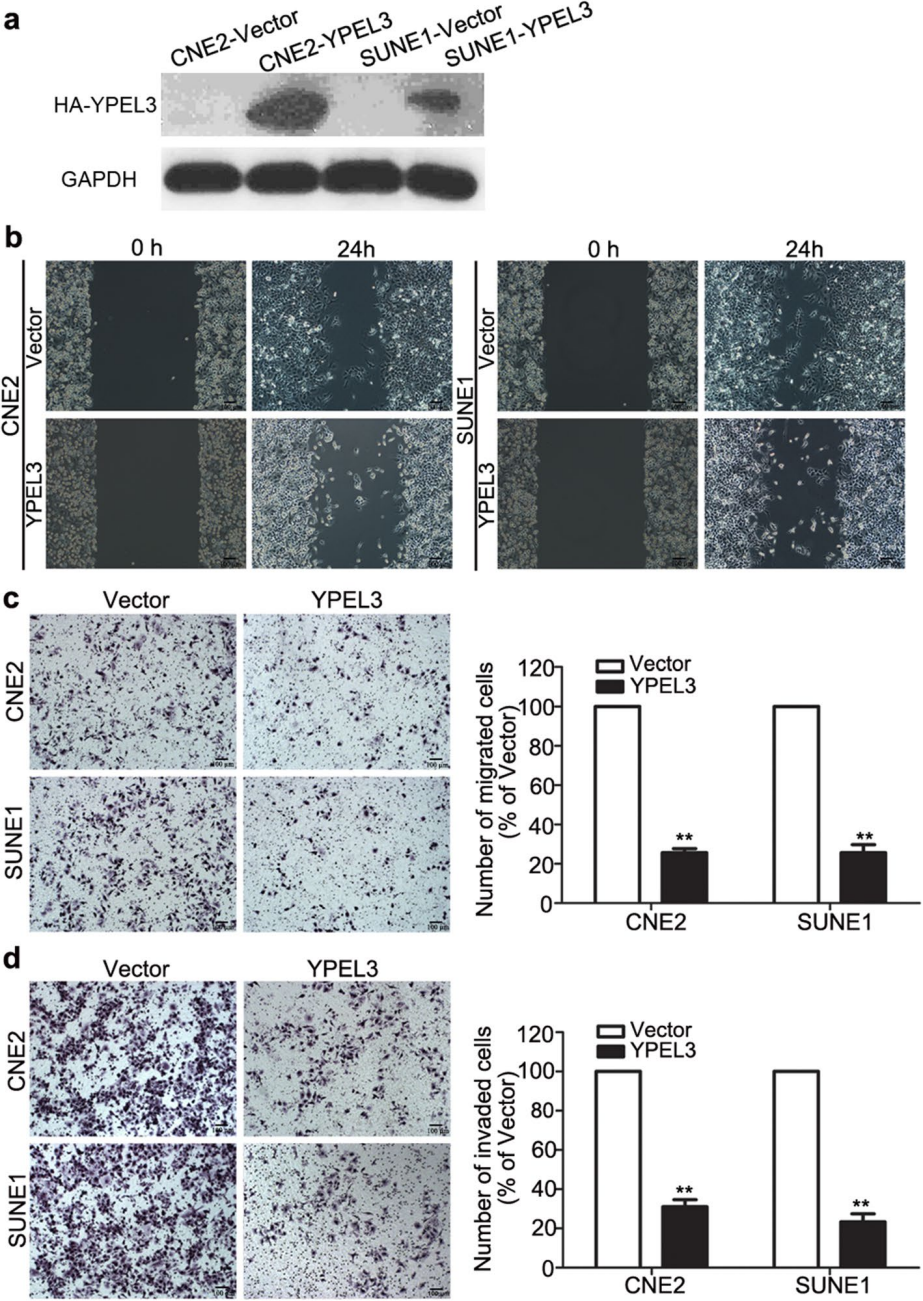
Effects of YPEL3 overexpression on NPC cell migration and invasion in vitro. **a** Representative western blotting analysis of YPEL3 overexpression in CNE-2 and SUNE-1 cells. GAPDH served as the loading control. **b-d** Representative images and quantification of the effects of YPEL3 overexpression on the migratory and invasive abilities of CNE-2 and SUNE-1 cells as determined by wound healing (**b**), Transwell migration (**c**), and invasion (**d**) assays. All of the experiments were performed at least three times. Data presented are the mean ± SD; **P < 0.01 compared with control using Student t-test

**Fig. 3:**
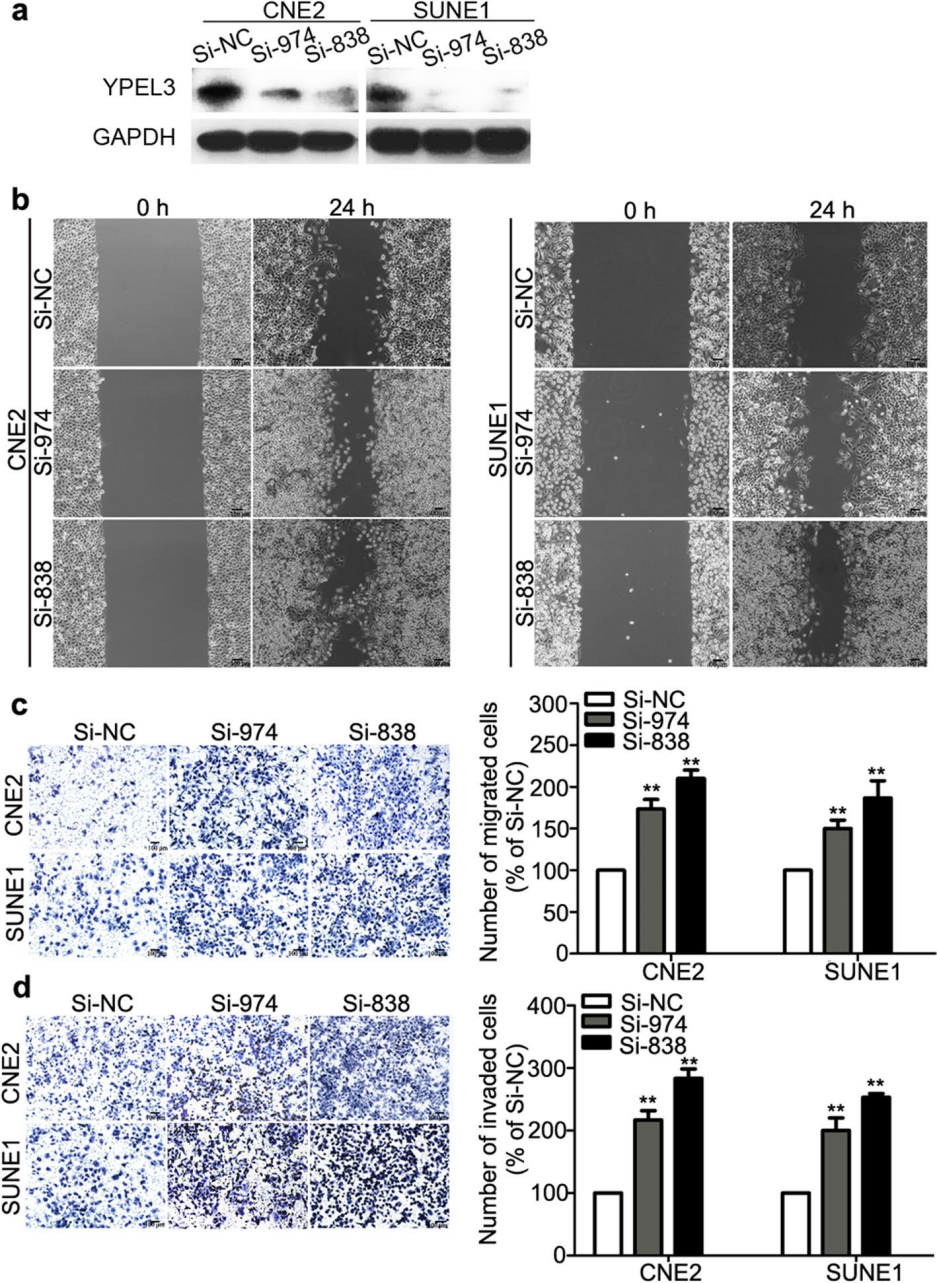
Effects of YPEL3 silencing on NPC cell migration and invasion in vitro. **a** Representative western blotting analysis of YPEL3 silencing in CNE-2 and SUNE-1 cells. GAPDH served as the loading control. **b-d** Representative images and quantification of the effects of YPEL3 silencing on the migratory and invasive abilities of CNE-2 and SUNE-1 cells as determined by wound healing (**b**), Transwell migration (**c**), and invasion assays (**d**). All of the experiments were performed at least three times. Data presented are the mean ± SD; **P < 0.01 compared with control using Student t-test

**Fig. 6:**
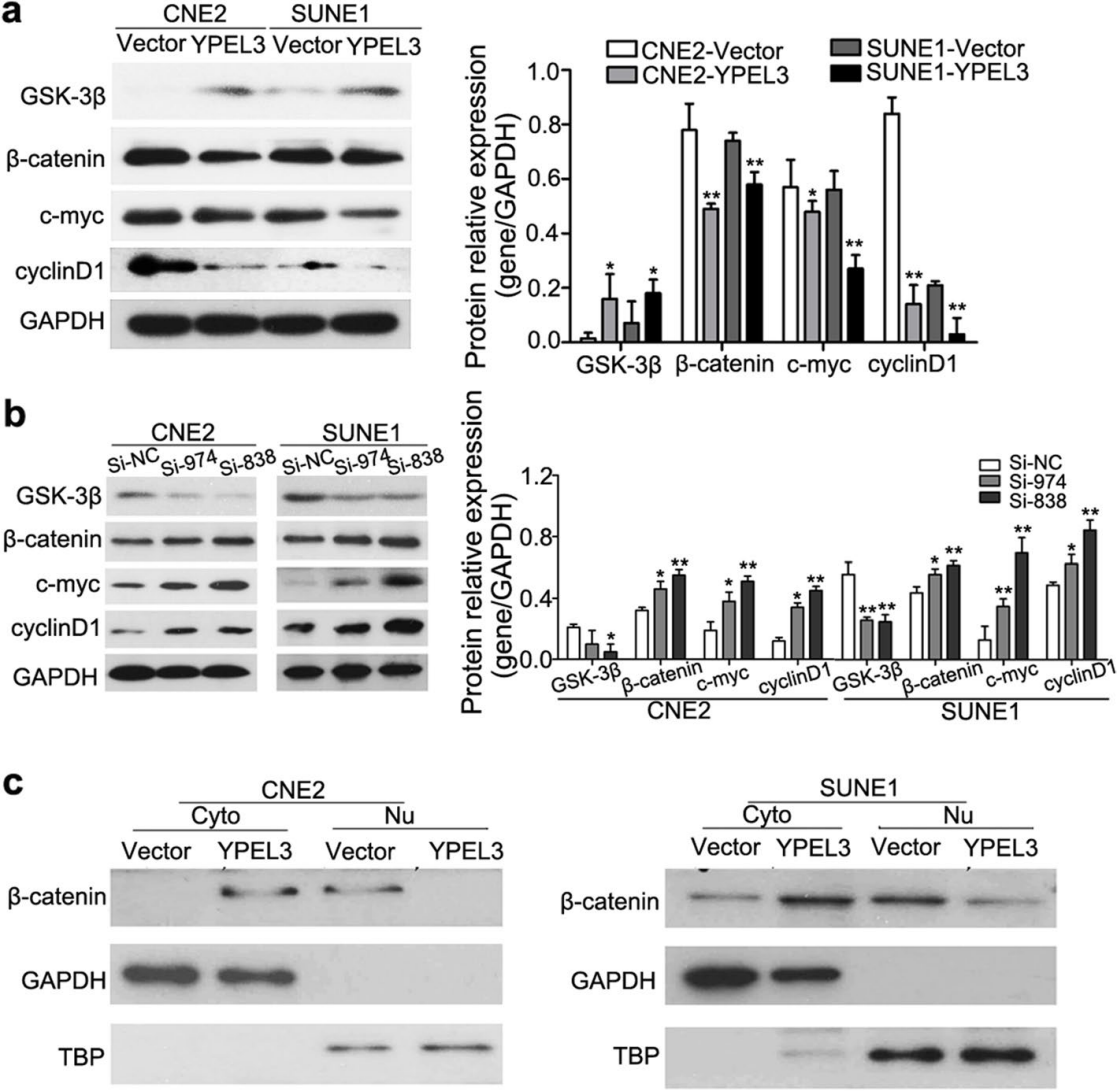
YPEL3 inhibited the Wnt/β-catenin signaling pathway. **a** Representative western blotting and quantification analysis of GSK-3β, β-catenin, c-MYC, and cyclin D1 expression levels after YPEL3 overexpression. **b** Representative western blotting and quantification analysis of GSK-3β, βcatenin, c-MYC, and cyclin D1 expression levels after YPEL3 silencing. **c** YPEL3 inhibited the nuclear (Nu) translocation of β-catenin. Cyto, cytoplasmic. All of the experiments were performed at least three times. Data presented are the mean ± SD; *P < 0.05 and **P < 0.01 compared with control using Student t-test

The corrected figure is given here. The correction does not affect the conclusions of the article. The original article has been updated.
